# Is There a Predisposition Gene for Ewing's Sarcoma?

**DOI:** 10.1155/2010/397632

**Published:** 2010-03-15

**Authors:** R. L. Randall, S. L. Lessnick, K. B. Jones, L. G. Gouw, J. E. Cummings, L. Cannon-Albright, J. D. Schiffman

**Affiliations:** ^1^Sarcoma Services, Department of Orthopaedics, Huntsman Cancer Institute and Primary, Children's Medical Center, The University of Utah, Utah, UT 84112, USA; ^2^Department of Oncological Sciences, Division of Pediatric Hematology/Oncology, Center for Children's Cancer Research, Huntsman Cancer Institute, The University of Utah, Utah, UT 84112, USA; ^3^Division of Medical Oncology, The University of Utah, Utah, UT 84112, USA; ^4^Department of Orthopaedics, Indiana University, Indiana, IN 46202, USA; ^5^Division of Genetic Epidemiology, Department of Internal Medicine, The University of Utah, Utah, UT 84112, USA; ^6^George E. Wallen Department, Veterans Affairs Medical Center, Salt Lake City, The University of Utah, Utah, UT 84148, USA

## Abstract

Ewing's sarcoma is a highly malignant tumor of children and young adults. The molecular mechanisms that underlie Ewing's Sarcoma development are beginning to be understood. For example, most cases of this disease harbor somatic chromosomal translocations that fuse the *EWSR1* gene on chromosome 22 with members of the ETS family. While some cooperative genetic events have been identified, such as mutations in *TP53* or deletions of the *CDKN2A* locus, these appear to be absent in the vast majority of cases. It is therefore uncertain whether EWS/ETS translocations are the only consistently present alteration in this tumor, or whether there are other recurrent abnormalities yet to be discovered. One method to discover such mutations is to identify familial cases of Ewing's sarcoma and to then map the susceptibility locus using traditional genetic mapping techniques. Although cases of sibling pairs with Ewing's sarcoma exist, familial cases of Ewing's sarcoma have not been reported. While Ewing's sarcoma has been reported as a 2nd malignancy after retinoblastoma, significant associations of Ewing's sarcoma with classic tumor susceptibility syndromes have not been identified. We will review the current evidence, or lack thereof, regarding the potential of a heritable condition predisposing to Ewing's sarcoma.

## 1. Introduction

The analysis of cancer predisposition syndromes has been an important approach towards the identification of oncogenes and tumor suppressor genes. Some hereditary cancer syndromes, such as Li-Fraumeni Syndrome, are caused by the mutation of critical tumor suppressor genes *(TP53) *and lead to wide-spread tumorigenesis including many different tumor types [1]. However, other hereditary cancer syndromes appear to have a more limited tumor spectrum.For example, individuals with syndromes such as WAGR (Wilms tumor, aniridia, genitourinary abnormalities, and mental retardation syndrome) and Denys-Drash Syndrome have mutations in the *WT1* gene, and these patients are primarily at risk for Wilms tumor [2, 3]. The identification of an underlying genetic mutation or predisposition to develop specific cancers is helpful not only to family members with that syndrome, but also to many other individuals who develop cancer without known risk factors. Knowledge of how specific tumors arise can be applied to targeted prevention, surveillance, and even therapeutic strategies.

Ewing's sarcoma, first described by James Ewing in 1921, is the second most common pediatric bone cancer after osteosarcoma. It is an aggressive cancer of children and young adults, with 30%–60% survival depending on tumor site and the presence or absence of metastases at diagnosis [4, 5]. While osteosarcoma is thought to arise from bone cell progenitors [6], the cell of origin of Ewing's sarcoma remains unknown. James Ewing himself initially described this disease as an endothelioma of bone, and later suggested that it arises from perivascular lymphatic endothelium [7, 8]. Since that time, other investigators have suggested myriad cells of origin, including hematologic [9], mesenchymal/fibroblastic [10, 11], and neural crest derivatives [12, 13]. More recently, emerging evidence has suggested that Ewing's sarcoma arises from a mesenchymal stem or progenitor cell [14–16]. A definitive answer to the cell of origin question will require additional analyses.

While the cell of origin of Ewing's sarcoma is not yet known, the molecular genetics of the tumor are better understood. Ewing's sarcomas are highly associated with a limited set of recurring, somatic chromosomal rearrangements. The most common of these, t(11;22)(q24;q12), is found in approximately 85% of cases, while t(21;22)(q22;q12) is found in 10% of cases [17, 18]. The remaining translocations are found in <5% each [19, 20]. These translocations fuse the *EWSR1* gene on chromosome 22 with an ETS family member, most commonly the *FLI1* gene on chromosome 11 [17, 19–21]. This Ewing's sarcoma-specific translocation generates an EWS/ETS fusion protein [17, 19–21]. The Ewing's sarcoma fusion proteins contain a strong transcriptional activation domain fused to an ETS type DNA binding domain and thus function as aberrant transcription factors that dysregulate target genes contributing to oncogenic transformation [22]. A number of genes that are dysregulated by EWS/FLI have been identified, and their roles in the oncogenic process are under active investigation [23–29]. The presence of EWS/ETS translocations is specific to Ewing's sarcoma, and the presence of an EWS/ETS fusion protein can be used clinically to diagnose patients with Ewing's sarcoma who have small round blue cell tumors.

Two main cooperating mutations have been identified in Ewing's sarcoma: p53 and RB pathway mutations [30–33]. Mutations in *TP53* (encoding the p53 protein) occur with a frequency of 5%–20% in Ewing's sarcoma, amplifications of *MDM2* occur in 0%–10% of cases, and deletions of the *CDKN2A* locus (encoding overlapping p16^INK4A^ and p14^ARF^ transcripts) occur in about 15% of cases [30, 32, 34, 35]. Thus, a significant percentage of Ewing's sarcoma have p53 pathway alterations. A similar percentage of Ewing's sarcoma tumors also have alterations in the RB pathway [30, 32, 33]. Alterations in these pathways may be required to bypass a growth inhibitory effect mediated by the EWS/ETS fusion protein [36, 37]. Although alterations in the p53 and/or RB pathways may cooperate with EWS/ETS fusion proteins to induce Ewing's sarcoma, this disease is not traditionally considered to be a part of the Li-Fraumeni syndrome and has rarely been reported as a second tumor in patients with heritable retinoblastoma [38–42]. Ewing's sarcoma does not appear to be a component of other tumor susceptibility syndromes, either.

There are no well-documented environmental causes of this disease and only a handful of epidemiological studies have focused on Ewing's sarcoma. While Ewing's sarcoma is not common, with an incidence of about 3 per one million people under 20 years of age [43], it remains uniformly deadly when untreated. Ewing's sarcoma has a slightly higher incidence in males. Interestingly, Ewing's sarcoma has a strong predilection for Caucasians, being far more common in this population than in Asians and ten times more common than in those of African descent. This Caucasian predilection is true globally [38]. 

A molecular postulate has been proposed for the racial predilection noted: intron 6, near the molecular breakpoint region, is at least fifty percent smaller due to diminished interspersed repeat sequences (*Alu * elements) in about 10 percent of the African population [44].It is hypothesized that (*Alu *elements are preferential sites for genetic recombinations in cancer [45]. Beyond the observation of different rates by ethnicity, Ewing's sarcoma is considered to be nonfamilial, with no genetic lineage predisposition.

## 2. Search Strategy and Selection Criteria

We reviewed the English literature to find any evidence in the demographics and epidemiology of Ewing's sarcoma to suggest a familial predisposition. We considered cases of consanguinity and any onco-syndromic conditions that might imply a predisposition genotype. Our results are described below. 

### 2.1. Ewing's Sarcoma and Related Tumors

Additional tumors beyond classic Ewing's sarcoma have been found to have similar histologic and molecular phenotypes, including the specific t(11; 22) translocation. Ewing's sarcoma and another small round blue cell malignancy often seen in soft tissues, termed primitive neuroectodermal tumor (PNET), were found to not only have similar histologic features but also to contain the identical translocation in greater than 95% of cases [46]. PNET is approximately 10-fold less common than Ewing's sarcoma. Some investigators have used the term “Ewing's Sarcoma Family of Tumors” to encompass Ewing's sarcoma, PNET, as well as atypical Ewing's sarcoma and Askin tumor (Ewing's sarcoma of the chest wall). All of these tumor types harbor the identical t(11;22) translocation. Because of the consistent genetic lesion, we will continue to refer to this entire group as Ewing's sarcoma.

There are currently no known cancer syndromes of which Ewing's sarcoma tumors are included, and Ewing's sarcoma tumors do not seem to be associated with any other types of tumors either in pediatric or adult oncology.

### 2.2. Demographics and Epidemiology

Chronologically, ninety percent of cases occur in patients between 5 and 25 years of age. After age 25, it is relatively rare. About 25% of cases occur before age 10, while 65% arise between ages 10 and 20 years old. Approximately 10% of patients are older than 20 years when they are diagnosed. Boys and young men are affected more frequently than girls and young women. Males also do less well than females. The pelvis is the most common location, followed by the femur, tibia, humerus, and scapula. However, Ewing's sarcoma can be found in any part in the body. 

Several reports have highlighted the general association of Ewing's sarcoma and parental exposure to pesticides, solvents, and farming or agricultural occupation [47–51]. Hernia, both inguinal and umbilical have also been linked to Ewing's sarcoma [47, 48, 52, 53]. Valery et al. [53] surmised that Ewing's sarcoma and hernia have common embryologic neuroectodermal pathways. Interestingly, these cases arose in farming families perhaps suggesting some unknown environmental influence the link of the two entities [53]. At least one report discounts this association [54]. In a case-control study, Winn et al. [54] matched 208 Ewing's cases with one sibling control and one age matched regional population control. Although hernia was seen 6 times more frequently among Ewing's patients compared to regional controls, sibling controls experienced hernias with the same frequency as Ewing's patients. Reports of patient height and onset of pubertal growth have been varied with no clear or consistent pattern developing in their association with Ewing's sarcoma [55–61].

The ethnic epidemiology of Ewing's Sarcoma is fascinating as it uniquely follows racial boundaries, similar to Wilms' tumor. Ewing's Sarcoma has a strong predilection for Caucasians, being far more common than in Asians and those of African descent [44, 62–65]. Zucman-Rossi et al. have noted that intron 6, near the molecular breakpoint region, is at least fifty percent smaller due to diminished interspersed repeat sequences (*Alu* elements) in about 10 percent of the African population [44]. *Alu* elements are a type of SINE, or Short INterspersed Element, and are approximately 300 bp in length with approximately 1,000,000 copies in the human genome, accounting for approximately 10% of genetic material. *Alu* elements are a type of transposon. It is hypothesized that *Alu* elements are preferential sites for genetic recombinations in cancer [45]. Perhaps in families with Ewing's sarcoma probands that develop remote Ewing's sarcoma in their descendents, increased genomic predominance of *Alu* elements leads to subsequent rechimerization of EWS/FLI and related translocations. Large-scale studies on germline DNA from Ewing's sarcoma patients have yet to be performed which could support this hypothesis.

Most recently, Johnson et al. explored the association between parental age and risk of all childhood cancers [66].The previous studies had explored the association between advanced maternal and paternal age with congenital syndromes (including several which predispose to cancer) [67] and a handful of other reports had provided preliminary support of an association between older parental age and an increased risk of some childhood cancers [68, 69]. Johnson et al. [66] followed up on these investigations and performed a pooled analysis on 17,672 childhood cancer cases diagnosed during 1980–2004 and 57,9666 controls born during 1970–2004. Cancer and birth registry records from New York, Washington, Minnesota, Texas, and California were linked, and Johnson et al. calculated logistic regression for parental age and specific childhood cancers adjusting for sex, birth weight, gestational age, birth order, plurality, maternal race, birth year, and state. Johnson et al. report that older maternal age seemed to increase the risk for most common childhood cancers. Interestingly, Ewing's sarcoma was found to be associated with the highest risk of all childhood cancer subtypes in relation to a 5-year increase in both maternal age (Odds Ratio 1.18 [1.02–1.35]) and paternal age (Odds Ratio 1.19 [1.06–1.34]). They speculate that the increased risk of cancer in older mothers could be due to age-related increases in de novo epimutations in oocyte genes transmitted to offspring [66]. A similar phenomenon in epimutations could be occurring in the spermatocytes of older fathers. Although limited to a single pooled analysis, this large study provides intriguing data to suggest a slight but possible contribution of genetic risk to the development of Ewing's sarcoma.

### 2.3. Possible Consanguinity

The association between Ewing's sarcoma and other forms of cancer seen in a proband's pedigree has been reported [70], some as early as 1952 [71]. Reporting on the Mayo clinic experience with Ewing's, McCormack et al. [71] noted that 9 of 80 patients (11%) were noted to have close family relatives, usually a grandfather or aunt, with some form of malignant tumor. In their series, only 1 patient had a sibling who had experienced a bone sarcoma. Eight years later the first reported incidence of Ewing's sarcoma in siblings was reported by Huntington et al. [72]. Two sisters, each diagnosed in their teens, eventually died of metastatic disease. None of their other siblings (five boys and two girls) showed any evidence of disease. A second report of Ewing's sarcoma in siblings was published in 1964 [73]. Hutter et al. [73] reported the case of two siblings, both female. One sister was diagnosed at age 3 and died of metastatic disease shortly thereafter. Her sister was diagnosed at age 16 and at the time of reporting was alive and disease free. Interestingly, their mother was treated for breast carcinoma, and their maternal grandfather died of carcinoma of the colon. Joyce et al. [74] reported the third case of Ewing's sarcoma in siblings in 1983. The first sibling was diagnosed at age 9 and treated successfully with chemotherapy and radiation. Her sister was diagnosed at age 19 and at the time of publication was alive with pulmonary disease that seemed responsive to chemotherapy. A careful history showed no reports of neoplastic disease in the immediate or extended family. 

Although isolated to case reports, these siblings with Ewing's sarcoma also would imply a slight but definitely suggestible contribution of genetics to the risk of developing Ewing's sarcoma. However, given their limited numbers and lack of genomic DNA for analysis, environmental contributions also cannot be ruled out. It is also interesting to note that these isolated siblings with familial Ewing's sarcoma were all females.

### 2.4. Onco-Syndromic Considerations

Finally, several authors have reported on the association of Ewing's sarcoma after diagnoses and treatment for retinoblastoma [75, 76]. Spunt et al. [42] published on a cohort of 6 Ewing's patients diagnosed after treatment for various cancers including lymphoma, leukemia, Wilms tumor, and retinoblastoma. Cope et al. [40], via meta-analysis, found that while Ewing's has been reported after a number of different malignancies. Only the predominance of retinoblastoma prior to Ewing's differs dramatically from the low frequency of retinoblastoma among childhood cancers in the general population. In contrast, cancers other than retinoblastoma were proportionate to those in the general population.

### 2.5. Microsatellites and Ewing's Sarcoma Risk

The mechanisms by which oncogenic ETS fusion proteins, which are DNA-binding transcription factors, target genes necessary for tumorigenesis are not well understood. Gangwal et al. analyzed promoters of these target genes and described a significant overrepresentation of highly repetitive GGAA-containing elements (microsatellites) [77]. They also reported that EWS/FLI uses GGAA microsatellites to regulate the expression of target genes, and that the ability to do so depends on the number of consecutive GGAA motifs. Gangwal et al. speculated that these microsatellite polymorphisms may contribute to differences in individual and population susceptibility to Ewing's sarcoma, and that this may also be true of other diseases mediated by ETS transcription factors [78]. Most recently, this same group combined transcriptional analysis, whole genome localization data, and RNA interference knockdown to identify glutathione S-transferase M4 *(GSTM4)* as a critical EWS/FLI target gene in Ewing's sarcoma [25]. They found that the recurrent Ewing's sarcoma translocation t(11;22) directly binds and regulates *GSTM4* expression through the same GGAA-microsatellite described above. Higher *GSTM4* expression correlated with worse clinical outcome. Microsatellite sizes differ between individuals, and so in addition to possible genetic contribution to Ewing's sarcoma susceptibility, there may be inherited differences in Ewing's sarcoma therapeutic responses. Ewing's sarcoma case-control studies analyzing microsatellite size and frequency are now required to support these findings.

## 3. Future Investigation

The epidemiological evidence supports a slight but possible genetic contribution to the risk of developing Ewing's sarcoma. However, due to its rarity, many of these studies lack statistical power to definitely prove or disprove a genetic susceptibility to this sarcoma. There is no “smoking gun” to suggest an underlying cancer predisposition in the majority of cases of Ewing's sarcoma. Large-scale studies investigating the genetic epidemiology of Ewing's sarcoma are sorely needed to answer the question of genetic disease risk. This will only be accomplished through group consortia and multi-institutional collaborations.

## 4. Conclusion

Ewing's sarcoma remains a deadly form of cancer in children and young adults. Unique and specific molecular genetic events define the pathogenesis of this tumor. It arises within defined ethnic boundaries yet only sporadic consanguinity has been reported. Because of its rarity, a remote familiality may have evaded detection thus far. We believe that an in depth investigation into the genetic epidemiology of Ewing's sarcoma is required to see if a predisposition gene or set of genes might contribute to this deadly disease in some subtle manner. This will only be accomplished through a stringent analysis of existing Ewing's sarcoma registries or large population databases.
